# Environmental variables and machine learning models to predict cetacean abundance in the Central-eastern Mediterranean Sea

**DOI:** 10.1038/s41598-023-29681-y

**Published:** 2023-02-14

**Authors:** Rosalia Maglietta, Leonardo Saccotelli, Carmelo Fanizza, Vito Telesca, Giovanni Dimauro, Salvatore Causio, Rita Lecci, Ivan Federico, Giovanni Coppini, Giulia Cipriano, Roberto Carlucci

**Affiliations:** 1 Institute of Intelligent Industrial Technologies and Systems for Advanced Manufacturing, National Research Council, via Amendola 122/D-I, 70126 Bari, Italy; 2 Ocean Predictions and Applications Division, Centro Euro-Mediterraneo sui Cambiamenti Climatici, Lecce, Italy; 3 Jonian Dolphin Conservation, viale Virgilio 102, 74121 Taranto, Italy; 4 School of Engineering, University of Basilicata, viale Ateneo Lucano 10, 85100 Potenza, Italy; 5 Department of Computer Science, University of Bari, via Orabona 4, 70125 Bari, Italy; 6 Department of Biology, University of Bari, via Orabona 4, 70125 Bari, Italy

**Keywords:** Ecology, Machine learning

## Abstract

Although the Mediterranean Sea is a crucial hotspot in marine biodiversity, it has been threatened by numerous anthropogenic pressures. As flagship species, Cetaceans are exposed to those anthropogenic impacts and global changes. Assessing their conservation status becomes strategic to set effective management plans. The aim of this paper is to understand the habitat requirements of cetaceans, exploiting the advantages of a machine-learning framework. To this end, 28 physical and biogeochemical variables were identified as environmental predictors related to the abundance of three odontocete species in the Northern Ionian Sea (Central-eastern Mediterranean Sea). In fact, habitat models were built using sighting data collected for striped dolphins Stenella coeruleoalba, common bottlenose dolphins Tursiops truncatus, and Risso’s dolphins Grampus griseus between July 2009 and October 2021. Random Forest was a suitable machine learning algorithm for the cetacean abundance estimation. Nitrate, phytoplankton carbon biomass, temperature, and salinity were the most common influential predictors, followed by latitude, 3D-chlorophyll and density. The habitat models proposed here were validated using sighting data acquired during 2022 in the study area, confirming the good performance of the strategy. This study provides valuable information to support management decisions and conservation measures in the EU marine spatial planning context.

## Introduction

The Marine Strategy Framework Directive (MSFD), Marine Spatial Planning (MSP) and Common Fisheries Policy (CFP) constitute the main policies to maintain the productive, resilient, and good health status (GES) of marine habitats to provide ecosystem services and limit the loss of biodiversity in EU Member States (EEA, 2015). This environmental strategy, although different in terms of achievable objectives, is based on the Ecosystem Based Management approach (EBM), which is assumed to be a holistic and integrated pathway worldwide. In particular, this approach aims to maintain or restore the composition, structure, function, and delivery of services of natural and modified ecosystems to achieve sustainability (Millennium Ecosystem Assessment, 2005). In this light, knowledge of the spatiotemporal distribution and abundance of target species, as well as the extension of their critical habitats and their overlap with highly impacted areas strongly characterized by anthropogenic pressures, is essential, especially in aquatic ecosystems.

Although, on a global scale, the Mediterranean Sea is one of the most important hotspots for its richness in marine biodiversity^1–3^, it has been historically threatened by numerous anthropogenic pressures, such as the presence of commercial maritime and fishing activities, a growing urbanization mostly along coastal zones, and the occurrence of different sources of pollution, from chemical to acoustic^4,5^. In addition, climate change, the spreading of alien species, and the increasing occurrence of disease outbreaks are considered the most recent verified drivers of impact on the basin^6–8^. The cetaceans of the Mediterranean Sea are among species worthy of conservation distributed in a heavily anthropized basin^9^. In this context, dolphins and whales can be exposed to several impacts, such as bycatch, competition of resources due to fishing activities^10–14^, shipping collisions^15^, chemical pollution from persistent organic pollutants, marine litter, heavy metals^16–18^ and noise pollution^19,20^.

To date, several studies^21–32^ have been conducted to provide information on the estimated abundance and distribution of regular species occurring in Mediterranean eco-regions. Moreover, over the last three decades, extensive literature describing methodological approaches to assess the abundance of top marine predators has rapidly expanded^33–36^, starting from methodologies requiring basic information (e.g., number of individuals, distances, photoidentification data), based on distance sampling^37–39^ and mark-recapture methods^40–42^, to more advanced techniques requiring a greater amount of data^36^. The latter brings us face to face one of the big challenges in ecology, namely, the identification of environmental predictor variables, which help to forecast bioecological responses based on environmental changes^43–45^. An example of the modeling approaches for an abundance assessment are model-based estimation methods, such as density surface modeling^46^, species distribution modeling^47^, and the most powerful machine learning techniques^48^. Generalized Additive Models^49^, Neural Networks^50^, Least Squares Boosting^51^, Random Forest^52^ and Support Vector Machines^53^ are some of the most popular learning models and have already been successfully applied in several application domains^43,54–63^. Despite this plethora of information, the question regarding cetacean species abundance in the Mediterranean Sea is far from being closed. In fact, enormous efforts are required in the continuous updating of the collected data, thus covering longer periods of sightings of cetacean populations, as well as larger study areas. Moreover, the evolution of the theoretical and computational improvements of the modeling approaches for the estimation of abundance and for the identification of environmental predictors require a continuous training of the models through the employment of state-of-the-art statistical techniques and strategies.

This study starts with the identification of the main environmental predictors related to the abundance of three cetacean species: the striped dolphin *Stenella coeruleoalba*, the common bottlenose dolphin *Tursiops truncatus*, and the Risso’s dolphin *Grampus griseus*, observed in the Gulf of Taranto (Northern Ionian Sea, Central-eastern Mediterranean Sea). A group of 28 environmental variables, extracted by the Copernicus Marine Service (https://marine.copernicus.eu/it) and EMODnet-bathymetry dataset (https://www.emodnet-bathymetry.eu/data-products), are tested to train three regression models: RF, LSBoost, and NN. Next, estimates of species abundance are provided as approach examples of habitat suitability definitions and baselines for these odontocetes in the Mediterranean Sea. Habitat models were developed using sighting data collected during marine surveys in the study area from July 2009 to October 2021. Finally, the most important variables for building these habitat models were identified and examined, and a validation of the proposed strategy for abundance estimation was provided using sighting data collected in 2022. All the sighting data and environmental variables, used in this study, are freely available.

## Results

All data were analyzed using MATLAB (MathWorks, Natick, MA). To build habitat models, dataset D, which collected sighting data in the period 2009–2021, was used (see “Data description” section in the “Materials and methods”). In particular, abundance data from striped dolphin, common bottlenose dolphin and Risso’s dolphin have been analyzed in relation to the following geographical, physical, and biochemical ocean variables: latitude, longitude, distance of the sighting from the coastline (Distance_From_Coast), maximum depth of the water column in the numerical model (Max_Depth), bathymetry (Emodnet_Depth), primary production*, nitrate*, phosphate*, phytoplankton carbon biomass* (PHYC), 3D-chlorophyll* (CHL3D), chlorophyll a (CHLA) at surface (CHLA), temperature*, salinity*, density*, mixed layer depth, thermocline depth (depthOfMaxN2), currents speed* (Currents_Intensity) and direction* (Currents_Direction). The three-dimensional variables have been labeled with * symbols and contain two values: the *top* value, which is the value computed at the top of the water column. corresponding to the variable mean value in the range [0, 40 m]; the *bottom* value computed at the bottom of the water column, given by the variable mean value in [50 m, 200 m]. Hence, a total of 28 environmental variables were used to train the models.

The performances of three regression models, LSBoost, RF and NN, in terms of Root Mean Square Error (RMSE), have been evaluated using a K-fold cross-validation, with K empirically set equal to five. Therefore, each model was trained on 80% of the available examples in the dataset D and tested on the remaining 20% at each run of the cross-validation procedure, and evaluation metrics were obtained by averaging values in the five runs. Parameter tuning for each model was empirically performed (see the “Regression models” section of the “Materials and methods”). The first experiment was conducted on the sighting data of striped dolphin (S), common bottlenose dolphin (T), and Risso’s dolphin (G). The performances of the three models, whose optimized.

hyperparameters are shown in Supplementary material, Table S1, were quite similar (see Table 1).

**Table 1:** Results of regression models LSBoost, RF, and NN.

Dataset	LSBoost	RF	NN
S	47	**46**	47
T	6	6	6
G	9	**8**	10

The performances of the models were evaluated in terms of RMSE trained on striped dolphin S, common bottlenose dolphin T, and Risso’s dolphin G datasets using five runs of the cross-validation procedure. Bold characters indicate the best performance among all the models.

In particular, RF performances were slightly better than those of NN and LSBoost, with an RMSE of 6 individuals for the T dataset, 8 for the G dataset and 46 for the S dataset. Very important is that RF required a lower training time; hence, in the following, we always refer to this algorithm.

**Figure 1:**
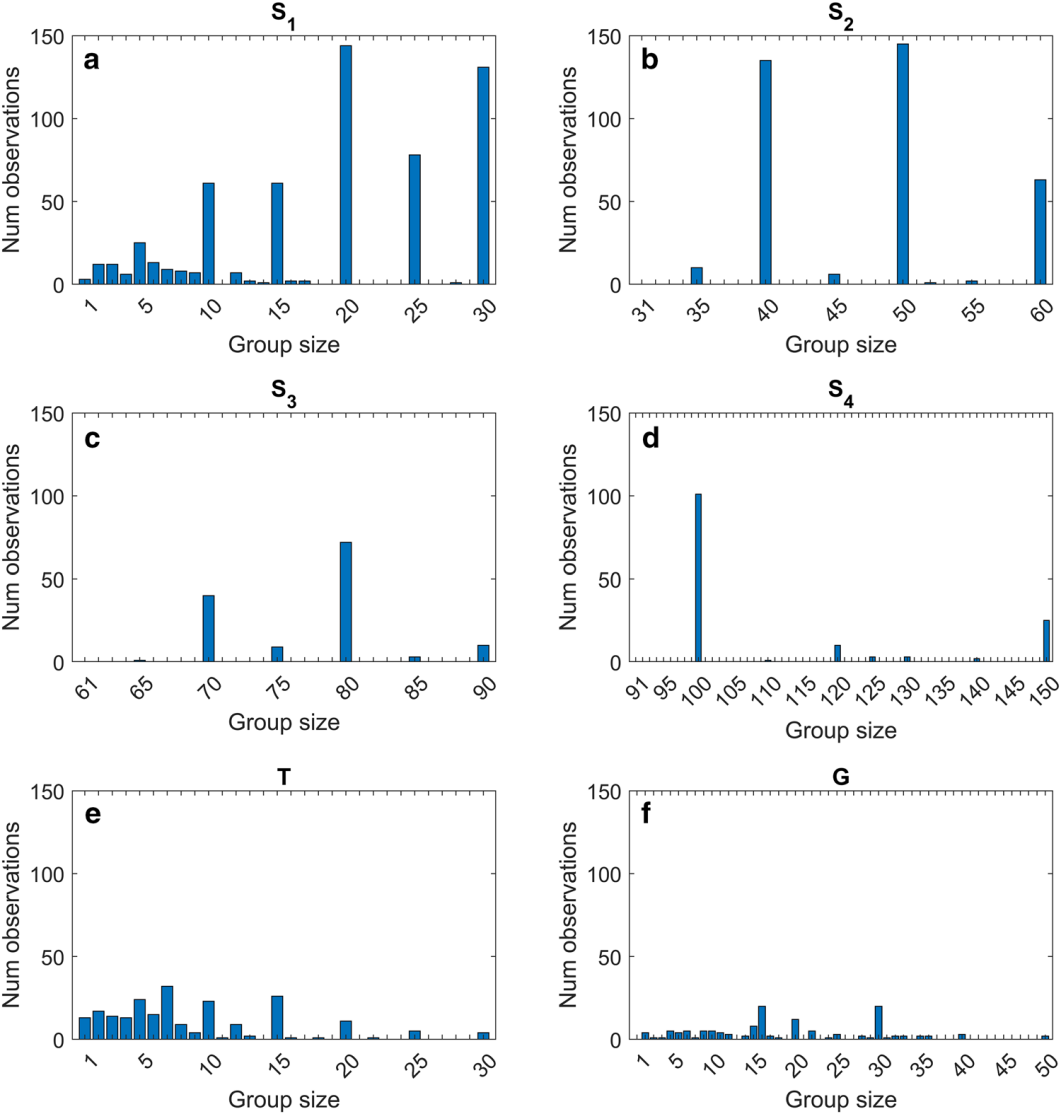
Sample distribution for datasets S_1_ (**a**), S_2_ (**b**), S_3_ (**c**), S_4_ (**d**), G (**e**) and T (**f**).

Differences between the results obtained on the T and G datasets with the results on the S dataset are reasonably due to multiple factors. Note that comparisons between datasets or models are out of the scope of this study. A first consideration is that the influence of the 28 variables on the prediction of cetacean abundance, made by RF models, could vary in different ranges of the values of group size, especially when these values vary greatly, thus influencing the performance of the regression model. Moreover, a main concern is the relatively minor dimension of groups of common bottlenose dolphins and Risso’s dolphins with respect to those of striped dolphins. In fact, in the T and G datasets, the maximum number of individuals observed in the groups size during the sightings is equal to 30 and 50 individuals, respectively (see the “Data description” section in the “Materials and methods”), so marine mammal observers can be obviously more accurate when counting. Instead, striped dolphin group size values can reach even hundreds of individuals, making their counting very difficult when group size increases. Obviously, the introduced bias increases with the dimension of the group. Therefore, when the group size counting is inaccurate, the regression model will be trained on wrong data, affecting the value of the RMSE computed. To test this hypothesis, the dataset S has been split into four subsets, according to the size of the observed groups during the sightings: S_1_ contains 585 sightings whose group size ranges from 1 to 30; S_2_ counts 362 sightings with group size varying in the interval [31, 60]; S_3_ contains 135 sightings whose group size ranges from 61 to 90; S_4_ counts 145 sightings with group size in the interval [91, 150] (see Table 2 and Fig. 1).

**Table 2:** Results of RF analysis in terms of RMSE obtained on the subsets of S.

Subset	Group size	Number of examples	RMSE
S_1_	[1, 30]	585	9
S_2_	[31, 60]	362	7
S_3_	[61, 90]	135	6
S_4_	[91, 150]	145	19

The performances of the RF models were evaluated in terms of RMSE trained on the S1, S2, S3, and S4 datasets using five runs of the cross-validation procedure.

Dataset S contains only 37 sightings with group sizes greater than 150, whose values fall into very few bins (see Supplementary material, Fig. S1); hence, these samples were discarded in the following analysis. The four subsets and relative cutoffs were selected considering the max group size values observed in the T and G datasets, which were 30 and 50 individuals, respectively. Moreover, some empirical considerations were made to ensure enough examples in each subset to train the models. Finally, RF models were trained on the four subsets, and their performances in terms of RMSE are shown in Table 2, while their optimized hyperparameters are shown in Supplementary material, Table S2. Experimental results highlight good performances of RF on subsets S_1_, S_2_, and S_3_, showing RMSE values varying from 6 to 9 individuals. Instead, the RMSE of RF on S_4_ increases up to 19. Figure 1 shows the distribution of the examples in the different group sizes for the S_1_, S_2_, S_3_, S_4_, G and T datasets. The main difference between S_4_ and the other datasets is that few examples in a few bins of the S_4_ histogram are available. It is reasonable that the shape of this histogram affects the RF regression performance, which becomes worse than others. The appearance of the S_4_ histogram can be imputable to inaccurate observations when individuals are greater than approximately one hundred, while the frequency of sighting striped dolphin groups with this number or greater number of individuals becomes low. The tested hypothesis is confirmed by these results, and the proposed strategy based on RF and the 28 environmental variables for the abundance estimation for these odontocetes is effective, mostly when the group size is lower than 90 individuals. In addition, the identification of the most common influential predictors, among the 28 environmental variables considered here, was assessed by ranking their importance given by the RF models (see Table 3 and Supplementary material, Figs. S2:S7) and analyzing the first ten positions of the ranked lists.

**Table 3:** Features importance given by the RF model on the S_1_, S_2_, S_3_, S_4_, G and T datasets.

Features	S_1_	S_2_	S_3_	S_4_	G	T	Frequency (%)
Lat	0.13	0.01	0.09			0.14	67
Lon	0.10		0.09				33
Distance_From_Coast	0.11	0.03					33
Max_Depth							0
Emodnet_Depth			0.08			0.15	33
Primary_Production_top	0.11					0.14	33
Primary_Production_bottom						0.17	17
Nitrate_top	0.13	0.01		9.94	0.88	0.21	**83**
Nitrate_bottom				4.48	0.77		33
Phosphate_top			0.08		0.91		33
Phosphate_bottom							0
PHYC_top		0.04	0.08	6.49	0.95	0.15	**83**
PHYC_bottom						0.15	17
*CHL3D_top*			0.08	4.80	0.77	0.16	67
CHL3D_bottom		0.03				0.13	33
CHLA							0
Temperature_top	0.12	0.02	0.09	4.65	1.36		**83**
Temperature_bottom		0.01		5.08	1.03		50
Salinity_top	0.11	0.02	0.11	8.95	0.97		**83**
Salinity_bottom	0.10			4.98			33
Density_top	0.10	0.04	0.11		1.08		67
Density_bottom				2.69	0.84		33
Mixed_Layer_Depth	0.10						17
Depth_of_max_N2							0
Currents_Intensity_top		0.03					17
Currents_Intensity_bottom				3.81		0.13	33
Currents_Direction_top			0.17				17
Currents_Direction_bottom							0

The importance scores of the top ten features, evaluated by RF models, are reported. The frequency is computed as the number of datasets in which the feature is among the top ten ranked ones over the total number of datasets analyzed (equal to 6). Bold characters correspond to the highest value of frequency.

Feature importance measures how variables influence the model when predicting the response. The influence of a predictor variable increases with the value of this measure. The idea underlying the feature importance computed by RF models is that if a variable is influential in prediction, then permuting its values should affect the model error; if a variable is not influential, then permuting its values should have little to no effect on the model error. Overall, nitrate, phytoplankton carbon biomass, temperature, and salinity, at the top of the water column, were included in 83% of the models, followed by latitude, top 3D-chlorophyll and top density in 67% of the models. Note that temperature at the bottom of the water column was also important in half of the models. Globally, the less influential variables (i.e., variables with zero frequency in the first ten positions of the ranked lists) resulted in the maximum depth, chlorophyll *a* at the surface, thermocline depth and bottom current direction and bottom phosphate.

In particular, the group size of striped dolphin seems to be positively influenced by the concentration of nitrate, especially for datasets S_1_ and S_4_, which is contrary to what was observed for T and G, for which this parameter seems negatively affects their group size although in a slight way (Supplementary material, Fig. S9). However, it is currently difficult to hypothesize about the meaning of these relationships, which need to be further investigated, also using larger sighting datasets. Additionally, the salinity and the temperature at the top layer positively affected the group size of this species, especially for dataset S_4_ and datasets S_1_ and S_2_, respectively (Supplementary material, Figs. S10 and S11). Similarly, the temperature at the top layer positively affected the group size of Risso’s dolphin, while the phytoplankton carbon biomass was negatively correlated with its group size (Supplementary material, Figs. S11 and S12). For the common bottlenose dolphin, in addition to the concentration of nitrates, which were found to negatively influence the size of the groups (Supplementary material, Fig. S9), other environmental features that were important for the prediction of abundance were primary production, 3D-chlorophyll and Emodnet_depth (Supplementary material, Fig. S7).

Finally, the proposed strategy for the cetacean abundance estimation was validated using 5 sighting data of striped dolphin and 2 of bottlenose dolphin collected in the study area during 2022, obtaining good performances with an average RMSE equal to 6 individuals.

## Discussion

Assessing the abundance of top marine predators and identifying the relationship between their abundance and environmental variables are primary goals in the framework of EU policies aimed to protect and preserve biodiversity and ecosystems^9,64–66^ for the adequate understanding of habitat suitability for different species and the implementation of correct conservation measures.

Here, we propose a modeling strategy that uses RF and a robust statistical methodology to estimate cetacean abundance and to identify the most influential environmental predictors. We tested and validated it using sighting data on three different cetacean species collected in the Gulf of Taranto over a span of over ten years. Although previous studies were conducted in the same area to predict the distribution and abundance of striped and common bottlenose dolphins as a function of environmental and anthropogenic drivers^22,24^, this study tested a high number of features that go beyond the “classic” physiographic or environmental variables, such as depth, distance from coast, slope, sea surface temperature and chlorophyll *a* content. This approach allowed us to verify the most powerful statistical method among the most innovative techniques of artificial intelligence to predict the group size of dolphin species according to several predictors and to investigate the importance of other environmental variables less used in the modeling.

The concentration of nitrate, phytoplankton carbon biomass, temperature and salinity, especially in the top layer of the water column, were frequently the most relevant features for the prediction of the group size of the three cetacean species investigated here. This result is somewhat expected because these variables are strongly linked to primary production and to the occurrence of prey (i.e.,^36,67,68^). The positive influence of the concentration of nitrate and salinity on the group size of striped dolphins is similar to those reported in the eastern tropical Pacific Ocean^69^, and a positive correlation between the temperature and the group size of striped dolphins was already reported in the Northern Ionian Sea^30^.

Similarly, the higher abundance of Risso’s dolphin in warmer waters converges with observations reported for the same species off the California coast^70,71^. Regarding the features that were significant in predicting the group size abundance of common bottlenose dolphins, such as primary production, the concentration of chlorophyll *a* and depth; these are commonly used features in several studies (i.e.,^72–74^) were positively correlated with the abundance and distribution of species except for depth. In particular, outcomes included in this study converge with results reported by Chavez-Rosales et al. in^36^, whose scope was to identify the main environmental covariates tied to the abundance of 17 cetacean species in the Western North Atlantic Ocean by using Generalized Additive Models (GAM). Top temperature was highly relevant in the habitat models for Risso's striped dolphins. In addition, primary production was important for bottlenose dolphins, as already found in the Western North Atlantic Ocean. In contrast, distance from the cost is one of the most common covariates in^36^, while it was influential only for small groups of striped dolphins, of less than 60 individuals, in the present study (see Supplementary material, Figs. S2 and S3). Considering the statistical approaches, to the best of our knowledge, GAM and RF are among the most powerful machine learning algorithms used to predict species abundance. There is an extensive body of literature confirming the predictive ability of GAMs for cetacean abundance estimation^75–77^, as well as an increasing interest in machine-learning techniques, such as RF^24,78^. A future aim will be to evaluate the most effective method for predicting cetacean species abundance. In machine learning framework it is a common practice to develop studies on the performance comparison of algorithms^79–82^, because these can provide meaningful insights into the research topic and can highlight direction to any future studies on that topic. In the modern literature, there are only a few previous studies on this matter^83^, and the subject is just as intriguing when considering other species; in fact, in^84^, the authors performed a comparison study between GAM and RF for the density estimation of two different bird species. However, despite this interest, the question needs to be further studied.

Sighting data used in our analysis covered an extended period, from 2009 to 2022. However, a limitation of this paper is that, unfortunately, despite the research effort, the number of available samples remains relatively small; in fact, only 129 sightings for Risso's dolphins and 225 for bottlenose dolphins are available. Instead, 1264 sightings of striped dolphins have been collected; however, in the present study we pointed out that it is convenient to divide this dataset into 4 smaller subsets (S1, S2, S3, and S4), according to the size of the observed groups during the sightings. The number of sightings collected in these four datasets varies from 135 to 585, and also in this case more samples are desirable for the further machine learning analysis. Moreover, the occurrence of group sizes in each dataset varies, with various elements with zero or very low number of available samples.

Another issue raised in this paper concerns the limitations in manually counting the number of individuals encountered by marine mammal observers. Developing innovative strategies based on Unmanned Aerial Vehicle or drones to support them in this task is strongly desirable and should be of great avail^85^. In addition, a great effort is needed when organizing and labeling data; this task can be time consuming and critical in the present field of application. A standardization of the expert labelling process of complex data, exploiting innovative approaches, is desirable and should be investigated in the future^86^.

Moreover, the proposed abundance estimation strategy shows good performance on sighting data collected in 2022, never seen before during model training. However, the validation set used here contains only 7 records. A future goal will be the further validation of the proposed strategy using a larger collection of data that will be acquired in the near future.

Last, the proposed strategy is general and could be effectively tested and applied to different geographical areas.

## Materials and methods

### Study area

**Figure 2:**
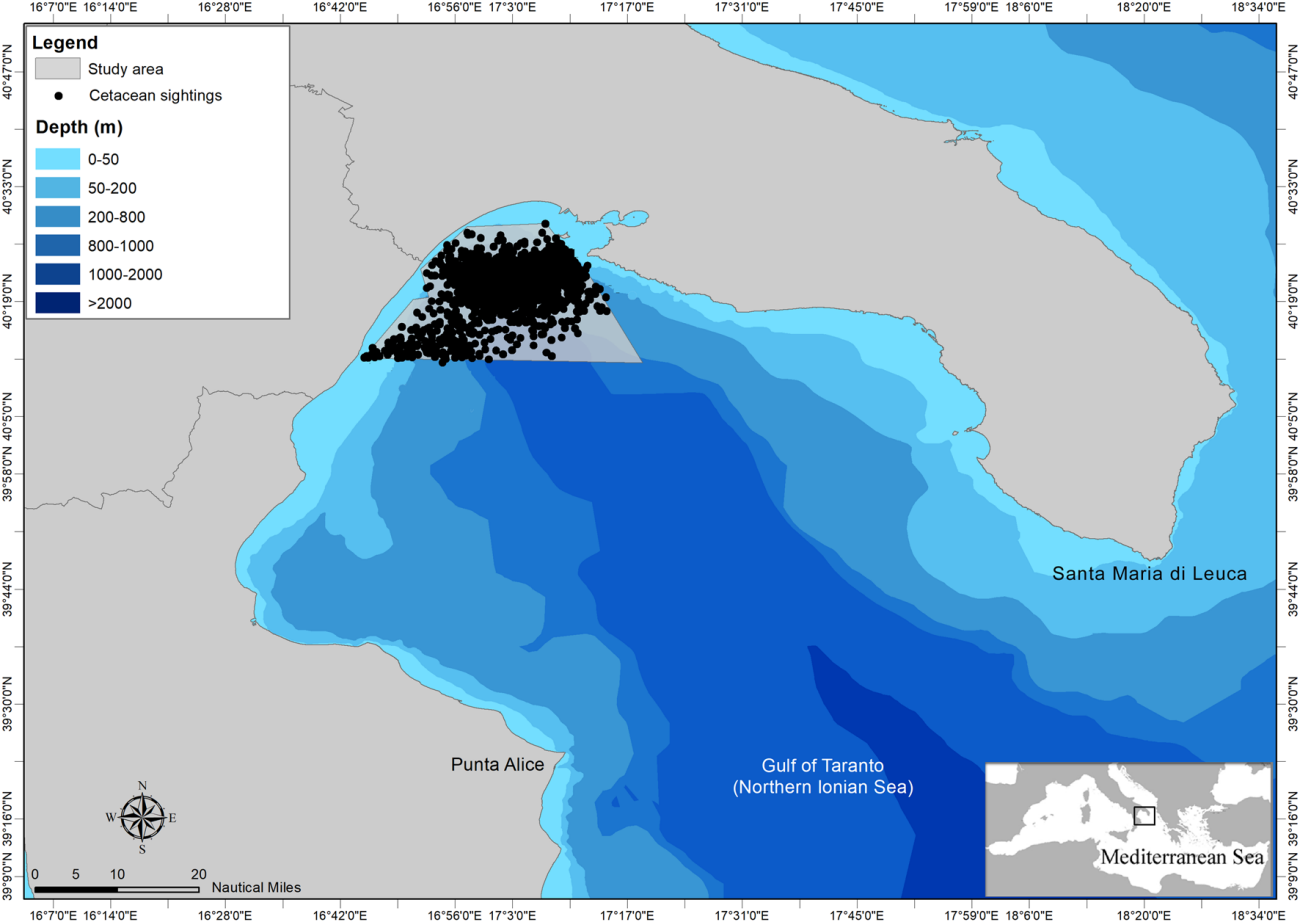
Map of the Gulf of Taranto (Northern Ionian Sea, Central-eastern Mediterranean Sea) with indication of the sightings and survey area investigated from 2009 to 2022.

The Gulf of Taranto is in the North-western Ionian Sea (Fig. 2). It is a semienclosed ocean area, covering approximately 14,000 km^2,22^ and includes the coasts of the Italian regions of Apulia, Basilicata, and Calabria. It is connected to the Northern Ionian Sea and the eastern Mediterranean Sea over an extended section (from Santa Maria di Leuca to Punta Alice), which includes a narrow trench deeper than 2000 m. The Gulf shows a very complex seabed topography characterized by descending terraces on the eastern side and by a narrow continental shelf with a steep slope and several channels on the western side. Centrally, the basin is characterized by the submarine canyon system of Taranto Valley with no clear bathymetric connection to a major river system^87–90^ (Fig. 2). The continental shelves—area shallower than 200 m—cover 10% of the total Gulf area. Wider shelves are present on the eastern side (Apulia), and five main rivers (Bradano, Basento, Agri, Sinni, and Crati) discharge from the western coastline with a relatively low annual mean runoff^91^. The morphology involves a complex distribution of water masses with a mixing of surface and dense bottom waters with the occurrence of high seasonal variability in upwelling currents^92–95^. From the oceanographic point of view, the basin-scale circulation of the Gulf is dominated by cyclonic gyres, with reversals in anti-cyclonic patterns occurring only 10–15 times for the period 1993–2018^91^. The ecosystem variabilities and changes (e.g., anomalous chlorophyll *a* bloom^96^) could be affected by the formation of downwelling/upwelling in the case of cyclonic/anti-cyclonic patterns. Possible coastal rim currents undergoing instabilities and forming submesoscale structures have been highlighted^97^. Furthermore, in^95^ authors described a mixed layer thickness extending down to 30 m during late summer, with an intermediate water salinity maximum—indicative of Modified Levantine Intermediate Waters—in the deep part of the Gulf. Authors in^98^ and in^99^ simulated the basin-scale and costal-scale circulation of the Gulf of Taranto using high-resolution models, highlighting the role of the Western Adriatic Coastal Current (WACC) position and strength^96,100–102^ in modulating the circulation patterns in the Gulf.

### Data description

Sighting data for striped dolphin, common bottlenose dolphin and Risso’s dolphin were collected from July 2009 to April 2022 during standardized vessel-based surveys carried out onboard a 12 m catamaran investigating an area of 960 km^2^. The sampling effort was set to approximately 5 h/day along 35 nautical miles (nm). Speed was maintained between 7 and 8 knots, and trips only occurred in favorable weather conditions (Douglas scale ≤ 3 and Beaufort scale ≤ 4). The scientific team onboard included three observers. The first was engaged in searching activity for targets at approximately 180°, while the others supported the activities of the former, searching in a sector from the track line to 90° on the starboard and port sides, respectively.

Once a target had been sighted, the dolphin group was switched to off-effort^38^, maintaining a minimum distance of approximately 50 m to avoid alteration in its behavioral activity. When the dolphins approached closer, the speed of the research vessel was reduced gradually until the engine was switched off. Sighting date, time of first contact, GPS position, group size and depth (m) were all recorded.

All sighting data are freely available (see Data Availability section) and have been divided into two parts: a dataset, named D, which collected data acquired in the period 2009–2021 and was devoted to training and testing regression models; and a validation set, which collected data from 2022 and was used to validate the models.

Dataset D contains 1618 records of cetacean sightings, with the following attributes: id, date, and position of sighting (latitude and longitude), number of sighted individuals and species (see Table 5). Among the 1618 records, 1264 are sightings of striped dolphin (denoted as S), 225 are sightings of common bottlenose dolphins (denoted ad T), and 129 records are sightings of Risso’s dolphins (denoted as G) (see Table 4).

**Table 4:** Dataset description.

Dataset	N	Min individuals	Max individuals	Mean individuals	Standard deviation of individuals
S	1264	1	500	50	48
T	225	1	30	9	6
G	129	2	50	19	10
Total	1618	–	–	–	–

N represents the number of sightings for each dataset; the minimum, maximum, mean, and standard deviation of the number of individuals recorded for each species are reported. S represents striped dolphin, T refers to common bottlenose dolphin and G to Risso’s dolphin.

**Figure 3:**
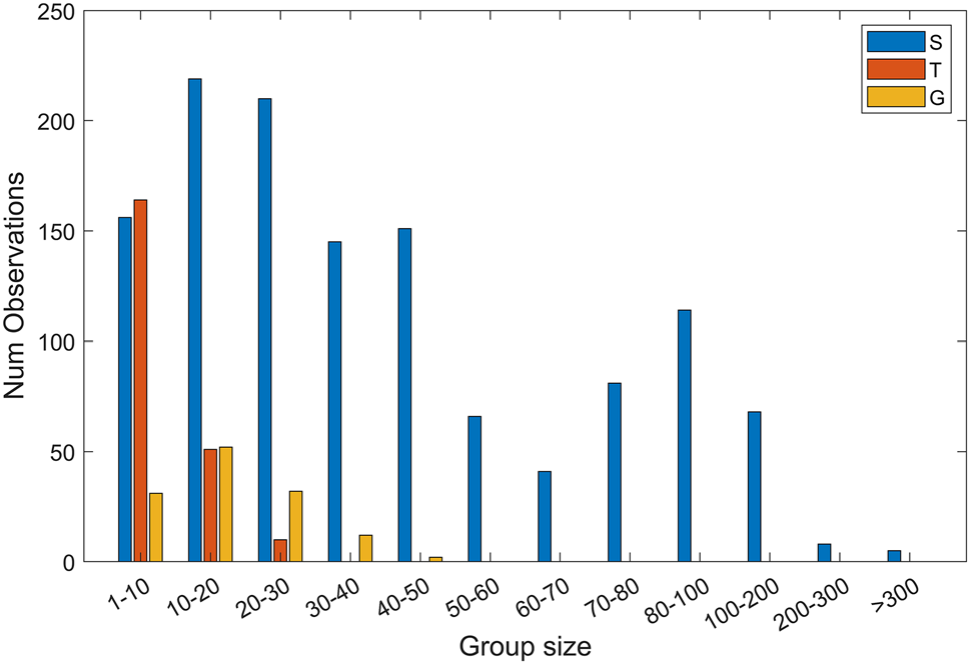
Distribution of the number of observations for different ranges of group sizes. The mark S refers to striped dolphin sightings, T to common bottlenose dolphin sightings and G refers to Risso’s dolphin sightings.

**Figure 4:**
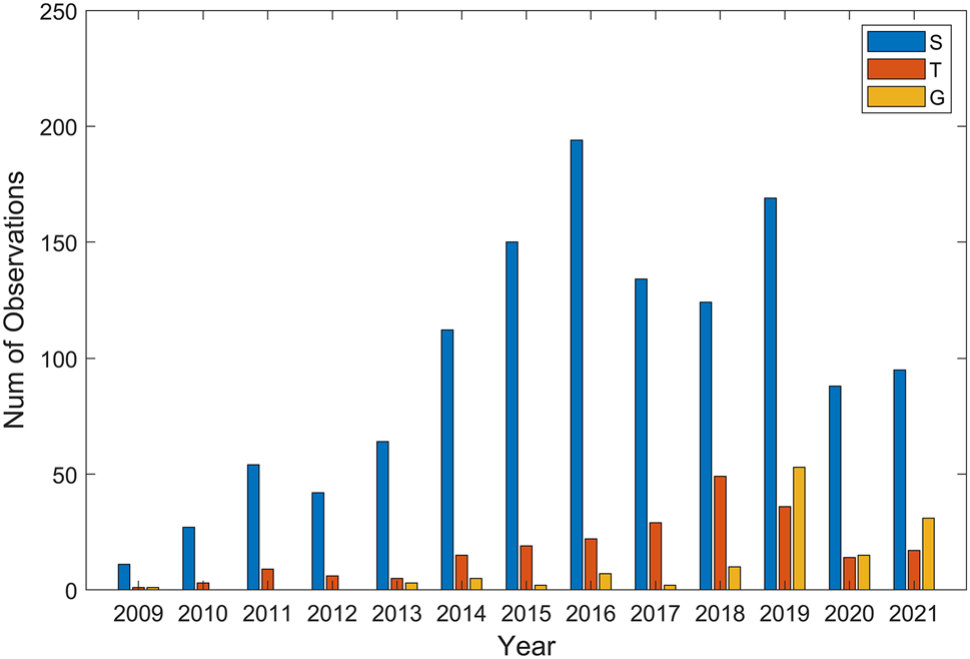
Distribution of the number of observations for the different species in the period 2009–2021: the mark S refers to striped dolphin sightings, T to common bottlenose dolphin sightings and G refers to Risso’s dolphin sightings.

Figure 3 illustrates the distribution of the number of observations among different group sizes in the S, G and T datasets. The different trends of the data in S compared with that of data collected in T and G are immediately evident; in fact, the group size in S reached higher values, up to 500 individuals, while in G and T, the maximum group size was equal to 50 and 30, respectively. The distribution of observations of S, T and G sightings in the period 2009–2021 is shown in Fig. 4.

Details on the seasonal distribution of these sightings data are shown in Supplementary material, Table S3.

The 1618 entries of the dataset D were enriched by:

- physical variables: ocean temperature, salinity, density, mixed layer depth, Brunt–Väisälä frequency, currents speed and direction;
- biogeochemical variables: primary production, nitrate, phosphate, phytoplankton carbon biomass, chlorophyll;
- auxiliary variables: max depth of the water column of the numerical model, high-resolution bathymetry, and distance of the sighting location from the coastline.

Table 5 shows a detailed description of the features used in this work. The physical features are provided by the Mediterranean Sea Physics reanalysis, produced by CMCC (IT)^103,104^ and delivered by Copernicus Marine Service. The product is generated by a numerical system composed of a hydrodynamic model, the Nucleus for European Modeling of the Ocean (NEMO,^105^), and a variational data assimilation scheme (OceanVAR,^106^). OceanVAR assimilates temperature and salinity vertical profiles and Sea Level Anomaly along satellite track data. The model horizontal grid resolution is 1/24° (ca. 4–5 km), and the unevenly spaced vertical levels are 141. In addition, the water density was computed according to^107^. The computation of the Brunt–Väisälä frequency (N2) was conducted using Copernicus Marine Service temperature and salinity, according to^108^.

**Table 5:** List of the 97 features used in this study.

Name	Description	Class	Source	Levels	Units	N	N’
Id	Id number of sighting	/	/	/	/	1	–
Data	Data of sighting	/	/	/	/	1	–
Specie	Sighted specie	/	/	/	/	1	–
Lat	Latitude	/	/	/	degree	1	1
Lon	Longitude	/	/	/	degree	1	1
Temperature*	Temperature	phy	Model	Surface, 10, 20, 30, 40, 50, 100, 200	°C	8	2
Salinity*	Salinity	phy	Model	Surface, 10, 20, 30, 40, 50, 100, 200	PSU	8	2
Density*	Density	phy	Computed- model	Surface, 10, 20, 30, 40, 50, 100, 200	kg/m^3^	8	2
Mixed_Layer_Depth	Mixed layer depth	phy	Model	/	m	1	1
N2*	Squared Brunt–Väisälä frequency	phy	Computed- model	Surface-10, 10–20, 20–30, 30–40, 40–50, 50–100, 100–200	cycle/h	7	1
Currents_Intensity*	Currents speed	phy	Model	Surface, 10, 20, 30, 40, 50, 100, 200	m/s	8	2
Currents_Direction*	Currents direction	phy	Model	Surface, 10, 20, 30, 40, 50, 100, 200	degree	8	2
Primary_Production	Primary production	bio	Model	Surface, 10, 20, 30, 40, 50, 100, 200	mg/m^3^/day	8	2
Nitrate*	Nitrate	bio	Model	Surface, 10, 20, 30, 40, 50, 100, 200	mmol/m^3^	8	2
Phosphate*	Phosphate	bio	Model	Surface, 10, 20, 30, 40, 50, 100, 200	mmol/m^3^	8	2
PHYC*	Phytoplankton carbon biomass	bio	Model	Surface, 10, 20, 30, 40, 50, 100, 200	mmol/m^3^	8	2
CHL3D*	3D-chlorophyll	bio	Model	Surface, 10, 20, 30, 40, 50, 100, 200	mg/m^3^	8	2
CHLA	Chlorophyll *a* at surface	bio	Satellite	Surface	mg/m^3^	1	1
Distance_From_Coast	Distance sighting-coastline	aux	Computed	/	km	1	1
Max_Depth	Maximum depth	aux	Model	/	m	1	1
Emodnet_Depth	Depth from EMODnet dataset	aux	EMODnet 2020 bathymetry	/	m	1	1
Total						97	28

The header specifies the variable Name and its long description header. The variable class distinguishes physical (phy), biogeochemical (bio) and auxiliary features. The source header describes the origin of the data (satellite, model, or computed from model data). The level header represents the depth at which data are provided. The column units contain the units of measurement. The Column N refers to the number of features available, while N’ refers to the number of variables used to train machine learning algorithms. The * symbol refers to three-dimensional variables.

The simulated biogeochemical features are provided by the Mediterranean Sea biogeochemical reanalysis, produced by OGS (IT) and delivered by Copernicus Marine Service^109^. The product at 1/24° horizontal resolution (ca. 4–5 km) is produced using the MedBFM3 model system. MedBFM3 includes the transport model OGSTM v4.0 coupled with the biogeochemical flux model BFM v5 and the variational data assimilation module 3DVAR-BIO v2.1 for surface chlorophyll. MedBFM3 is coupled offline with the physical reanalysis^103^, which provides daily forcing fields (i.e., currents, temperature, salinity, diffusivities, wind, and solar radiation). The ESA-CCI database of surface chlorophyll concentration (CMEMS-OCTAC REP product) is assimilated with a weekly frequency.

The chlorophyll *a* at the surface (CHL_A) observed from satellite is provided by the product Mediterranean Sea Reprocessed Surface Chlorophyll Concentration from Multi Satellite observations, produced by the Global Ocean Satellite monitoring and marine ecosystem study group (GOS) of the Italian National Research Council (CNR, IT^110^), within the Copernicus Marine Service. The Level-4 product includes the daily interpolated chlorophyll field with no data voids starting from the multi-sensor (MODIS-Aqua, NOAA-20-VIIRS, NPP-VIIRS, and Sentinel3A-OLCI) and the monthly averaged chlorophyll concentration for the multi-sensor and climatological fields, all at a 1 km resolution. Chlorophyll fields are obtained by means of the Mediterranean regional algorithms: an updated version of the MedOC4 (Case 1 waters,^111^, with new coefficients) and AD4 (Case 2 waters,^112^).

Among the auxiliary variables, high-resolution bathymetry was derived from the EMODnet-bathymetry dataset (2020-DTM (https://www.emodnet-bathymetry.eu/); the maximum depth of the water column (Max_Depth) was derived by the Mediterranean Sea Physics reanalysis; and the distance of the sighting location from the coastline (Distance_From_Coast) was computed using the geographical coordinates (lat, lon).

The features described above have been preliminarily processed before feeding the machine learning algorithms. First, the three-dimensional variables were extracted at the surface and at 10 m, 20 m, 30 m, 40 m, 50 m, 100 m, and 200 m. The variables have been limited up to 200 m of depth because of the stability and low variability of the water column below. Then, a sea-overland extrapolation procedure^99,113^ was used to prevent the presence of missing values interpolating the oceanic fields over each cetacean sightings record. This procedure uses a diffusive boundary layer approach that extrapolates the field values on the areas near the coastline where the Copernicus Marine Service solutions are not defined. The procedure iteratively computes the ocean quantities on the land grid points so that these quantities can be interpolated on the sighting records that are very close to the coast.

From the Brunt–Väisälä frequency N2, only one variable was derived, corresponding to the thermocline depth (i.e., depth of the max value of the N2 vector), entitled “depthOfMaxN2”, for each CMEMS grid point.

Following the temperature climatology in the study area during 2009–2021 (see Supplementary material, Fig. S8), primary production, nitrate, phosphate, phytoplankton carbon biomass, 3D-chlorophyll, temperature, salinity, density, current intensity and direction features were averaged in the intervals of [0–40 m] and [50–200 m]. Therefore, for each variable, two features were considered, named top when mean values are computed in the interval [0, 40 m] and bottom in [50–200 m].

Finally, the dataset, prescribed to the analysis with machine learning algorithms, included 1618 records of sightings, each enriched with the 28 variables (N’) previously described. The labels used to train the models were the number of individuals counted in each sighting.

Last, the validation set contains 7 records of cetacean sightings, of which 5 are of sightings of striped dolphins and 2 are of common bottlenose dolphins. For each record, the same 28 variables, already used for dataset D, were measured and used for models validation.

### Regression models

Random Forest^52^ is an ensemble method that uses multiple decorrelated decision trees that are merged to perform regression or classification tasks: each tree is built using a random subset of features and examples, while the results on the test set are obtained by computing the average of the results of each tree. LSBoost^51^ a variant of the Adaboost algorithm^58,114^, was used for the regression. Through a weighted combination of the outputs produced by a set of weak classifiers, LSBoost defines a function able to estimate the abundance of the dolphin groups. More specifically, at each step, the algorithm fits a new classifier to the difference between the observed response and the aggregated prediction of all classifiers grown previously. The aim is to minimize the mean-squared error. All new classifiers are fitted to ${y}_{n}-\upeta \mathcal{F}\left({x}_{n}\right)$, where ${y}_{n}$ is the observed response, $\mathcal{F}\left({x}_{n}\right)$ is the aggregated prediction from all weak classifiers grown thus far for observation ${x}_{n}$, and $\upeta$ is the learning rate.

**Figure 5:**
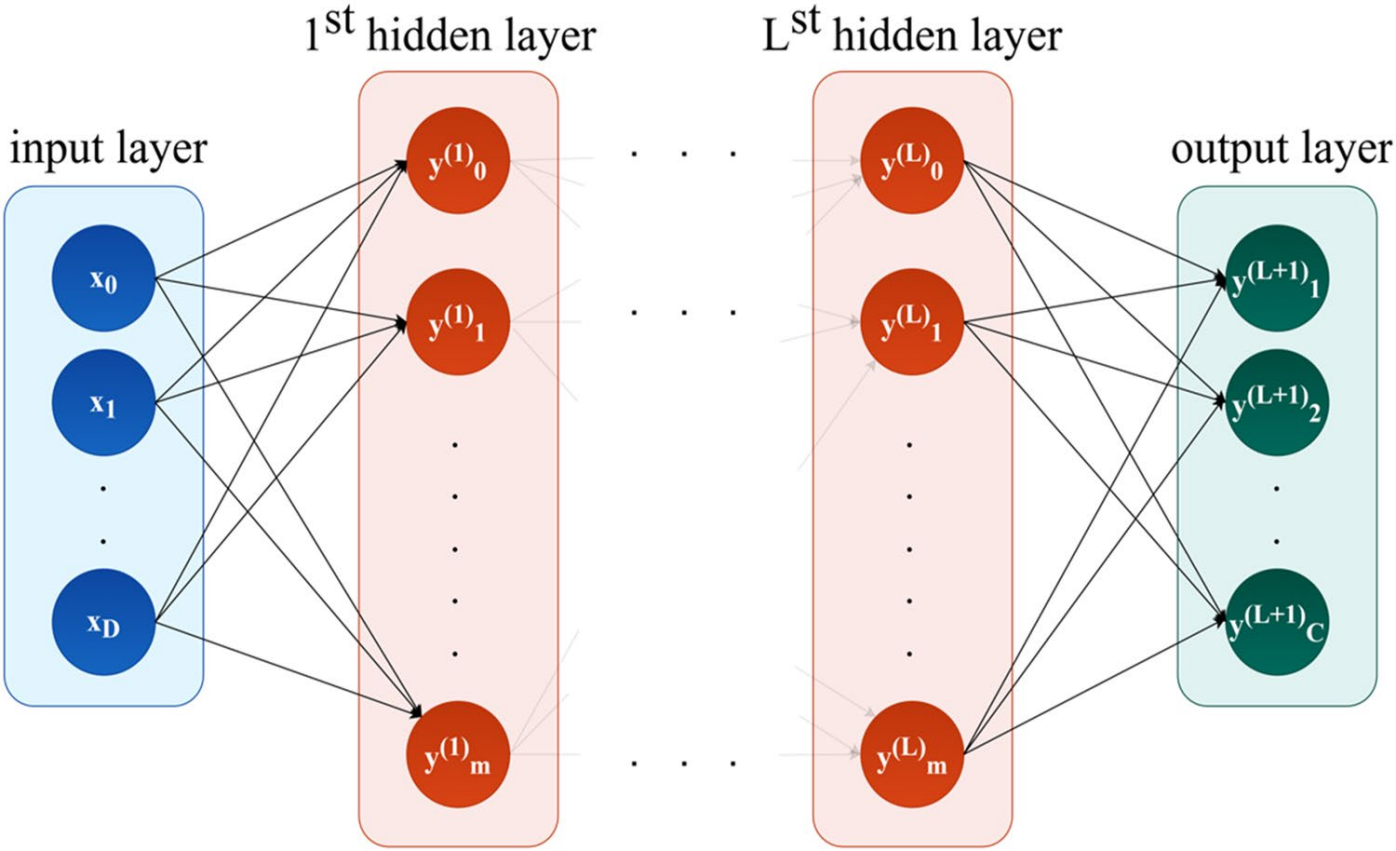
Feedforward fully connected neural network with (L + 1) layers with D input units and C output units.

Finally, to compare the traditional regression models with modern deep learning techniques, a feedforward fully connected NN was developed (see Fig. 5). This class of networks consists of multiple layers of computational units, usually interconnected in a feed-forward way^50^. Each neuron in one layer has directed connections to the neurons of the subsequent layer. RF, LSBoost and NN models have been trained with automatic parameter tuning using Bayesian optimization^115^. This means that the model settings are initialized as default; after a first full cross validation on the available dataset, these settings are updated according to the just obtained performance and the current learning rate; this process is iterated for a certain number of times or until the model converges. Of course, in the end, the settings that provided the best performance were considered. Bayesian optimization uses a surrogate for the objective function, which is much easier to optimize than the objective function. It works by finding the next set of hyperparameters to evaluate the actual objective function by selecting hyperparameters that perform best on the surrogate function. In our case, the optimizable parameters of the RF models are the maximum number of splits, minimum leaf size, numbers of predictors to sample and number of ensembles learning cycles^116^, while the optimizable parameters of the LSBoost models are the minimum leaf size, number of ensembles learning cycles, maximum number of splits and learning rate. Last, the optimizable parameters for the neural network models are the number of hidden layers, the size of each hidden layer, the activation function, and the regularization term strength. In our application, the number of hidden layers of the networks varied from 1 to 5, and for each layer, the number of neurons was in the range [1, 100]. The activation functions used in our analysis were ReLu, tanh, sigmoid and the identity function. The regularization term strength is optimized over continuous values in the range [1e^−5^,1e^5^]/(number of observations), where the value is chosen uniformly in the log transformed range.

### Root mean square error (RMSE)

The performance of a regression model is evaluated in terms of Root Mean Square Error (RMSE), a measure of the residuals between values predicted by a model and the values observed. It is defined as follows:

$$\mathrm{RMSE}=\sqrt{\frac{{\sum }_{i=1}^{N}{\left({y}_{i}- \widehat{{y}_{i}}\right)}^{2}}{N}}$$

where *N* is the number of samples, ${y}_{i}$ is the real estimation of the pod abundance and $\widehat{{y}_{i}}$ is the pod abundance predicted by the regression model.

## Supplementary Information

Supplementary Information.

## Data Availability

The datasets generated and analyzed during the current study are available on GitHub datarepository1/Environmental-variables-and-machine-learning-models-to-predict-cetacean-abundance repository at https://github.com/datarepository1/Environmental-variables-and-machine-learning-models-to-predict-cetacean-abundance. Further inquiries can be directed to the corresponding author.
