# Supplementary material for: Environmental variables and machine learning models to predict cetacean abundance in the Central-eastern Mediterranean Sea
Source: Sci Rep. 2023 Feb 14;13:2600. doi: 10.1038/s41598-023-29681-y (PMC9929343; doi:10.1038/s41598-023-29681-y)
Supplement: Supplementary file 1 — Supplementary Information. [file 41598_2023_29681_MOESM1_ESM.pdf]

Environmental variables and machine learning models to predict cetacean abundance in the Central-eastern Mediterranean Sea

Rosalia Maglietta[1], Leonardo Saccotelli[2], Carmelo Fanizza[3], Vito Telesca[4], Giovanni Dimauro[5], Salvatore Causio[2], Rita Lecci[2], Ivan Federico[2], Giovanni Coppini[2], Giulia Cipriano[6], Roberto Carlucci[6]*
(1) Institute of Intelligent Industrial Technologies and Systems for Advanced Manufacturing, National Research Council, via Amendola 122/D-I, 70126 Bari, Italy
(2) Ocean Predictions and Applications Division, Centro Euro-Mediterraneo sui Cambiamenti Climatici, Lecce, Italy
(3) Jonian Dolphin Conservation, viale Virgilio 102, 74121 Taranto, Italy
(4) School of Engineering, University of Basilicata, viale Ateneo Lucano 10 - 85100, Potenza, Italy
(5) Department of Computer Science, University of Bari, via Orabona 4, 70125 Bari, Italy
(6) Department of Biology, University of Bari, via Orabona 4, 70125 Bari, Italy
* corresponding author. e-mail to: rosalia.maglietta@cnr.it

Supplementary material

Table S1. Experiments were carried out on the sighting data of striped dolphin (S), common bottlenose dolphin (T), and Risso's dolphin (G), extracted from dataset D, using LSBoost, RF, and NN models. Optimized hyperparameters of regression models LSBoost, RF, and NN models for striped dolphin S, common bottlenose dolphin T, and Risso's dolphin G datasets, using automated parameters tuning. For both RF and LSBoost: MinLeafSize means the minimum leaf size; NumLearningCycles is the number of ensembles learning cycle; the maximum number of splits is called MaxNumSplits. For LSBoost, the learning rate is LearnRate, while for RF, NumVarToSample is the numbers of predictors to sample. Lastly, for NN NumLayers is the number of hidden layers, LayerSizes is the size of each hidden layer, ActivationFunction is the activation function, and Lambda is the regularization term strength.

Dataset	LSBoost	RF	NN
S	NumLearningCycles: 496<br>MinLeafSize: 109<br>LearnRate: 0.013<br>MaxNumSplits: 146	NumLearningCycles: 139<br>MinLeafSize: 20<br>NumVarToSample: 16<br>MaxNumSplits: 13	NumLayers: 5<br>LayerSizes: [1, 2, 2, 2, 100]<br>ActivationFunction: None<br>Lambda: 1.477
T	NumLearningCycles: 303<br>MinLeafSize: 1<br>LearnRate: 0.020<br>MaxNumSplits: 5	NumLearningCycles: 140<br>MinLeafSize: 1<br>NumVarToSample: 8<br>MaxNumSplits: 18	NumLayers: 5<br>LayerSizes: [1, 21, 7, 24, 2]<br>ActivationFunction: Tanh<br>Lambda: 0.001
G	NumLearningCycles: 312<br>MinLeafSize: 6<br>LearnRate: 0.016<br>MaxNumSplits: 30	NumLearningCycles: 106<br>MinLeafSize: 7<br>NumVarToSample: 10<br>MaxNumSplits: 127	NumLayers: 5<br>LayerSizes: [3, 2, 18, 62, 83]<br>ActivationFunction: ReLu<br>Lambda: $8.972e^{-07}$

Table S2. Optimized hyperparameters of RF models for striped dolphin S_1, S_2, S_3, S_4 datasets, using automated parameters tuning. Optimized hyperparameters of RF for datasets S_1, S_2, S_3, S4, using automatic parameters tuning are shown: MinLeafSize means the minimum leaf size; NumLearningCycles is the number of ensembles learning cycle; the maximum number of splits is called MaxNumSplits; NumVarToSample is the numbers of predictors to sample.

Subset	Optimized hyperparameters
S_1	NumLearningCycles: 51<br>MinLeafSize: 1<br>NumVarToSample: 9<br>MaxNumSplits: 27
S_2	NumLearningCycles: 31<br>MinLeafSize: 179<br>NumVarToSample: 2<br>MaxNumSplits: 12
S_3	NumLearningCycles: 498<br>MinLeafSize: 10<br>NumVarToSample: 3<br>MaxNumSplits: 18
S_4	NumLearningCycles: 22<br>MinLeafSize: 16<br>NumVarToSample: 26<br>MaxNumSplits: 28

Table S3. Summary of marine mammal sightings per season.

Specie	Total sightings per season / Total animals			
	Spring	Summer	Fall	Winter
Stenella coeruleoalba	308/17676	782/40055	141/5414	33/670
Tursiops truncatus	62/566	151/1305	8/75	4/23
Grampus griseus	17/139	107/2175	5/145	0/0

Specie	Total sightings per season / Total sightings			
	Spring	Summer	Fall	Winter
Stenella coeruleoalba	308/1264	782/1264	141/1264	33/1264
Tursiops truncatus	62/225	151/225	8/225	4/225
Grampus griseus	17/129	107/129	5/129	0/129

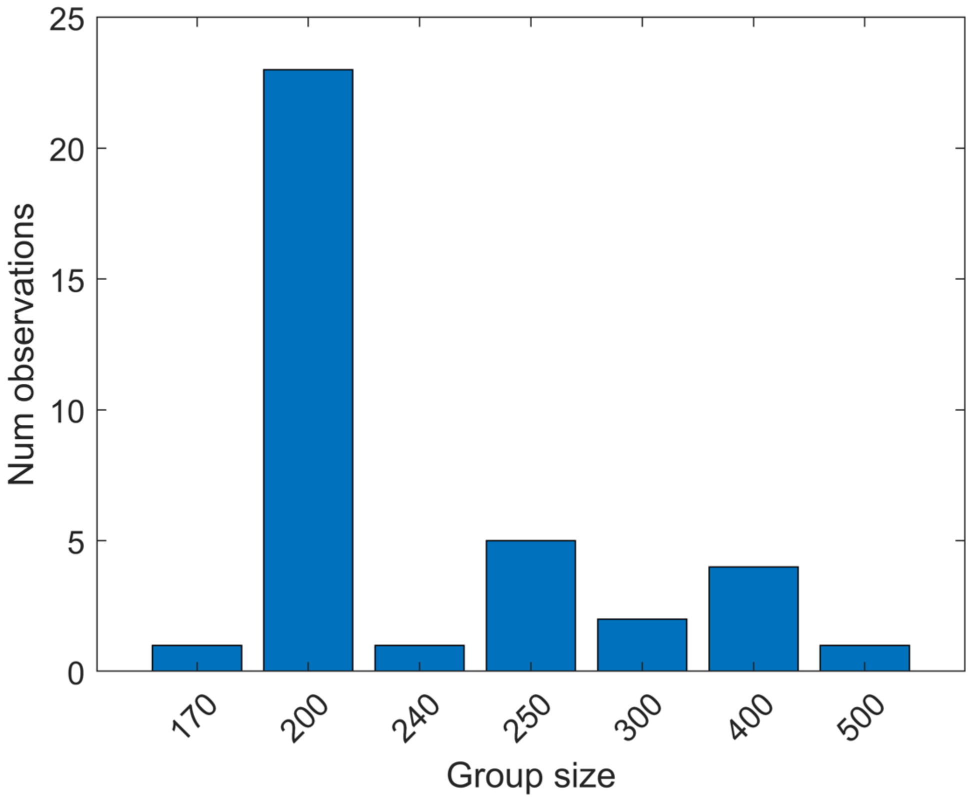

Figure S1. Samples distribution for the examples of dataset S, with group size greater than 150.

Details on Results The identification of the main environmental predictors was assessed by using the importance given by the RF models to the single features, results on S_1, S_2, S_3, S_4, G, and T datasets are shown in Figures S1:S6.

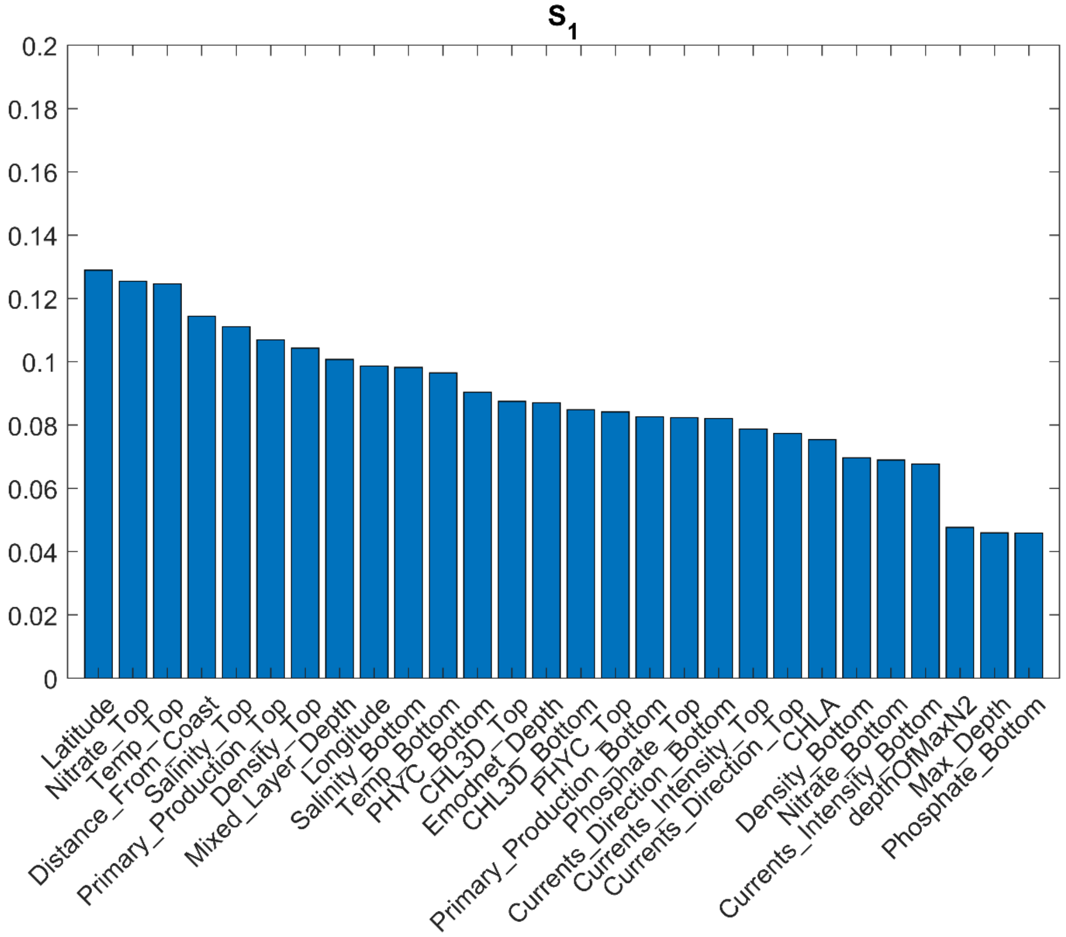

Figure S2. Features importance given by the RF model on dataset S_1.

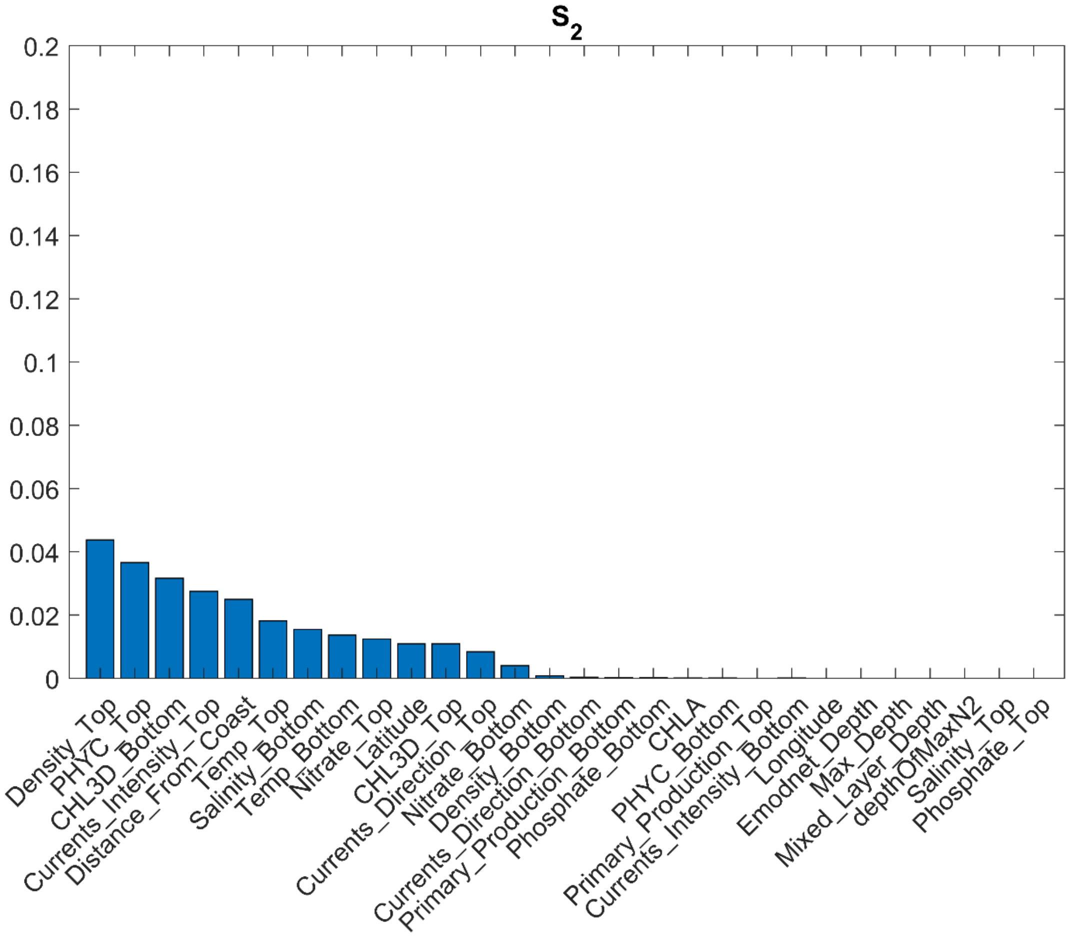

Figure S3. Features importance given by the RF model on dataset S_2.

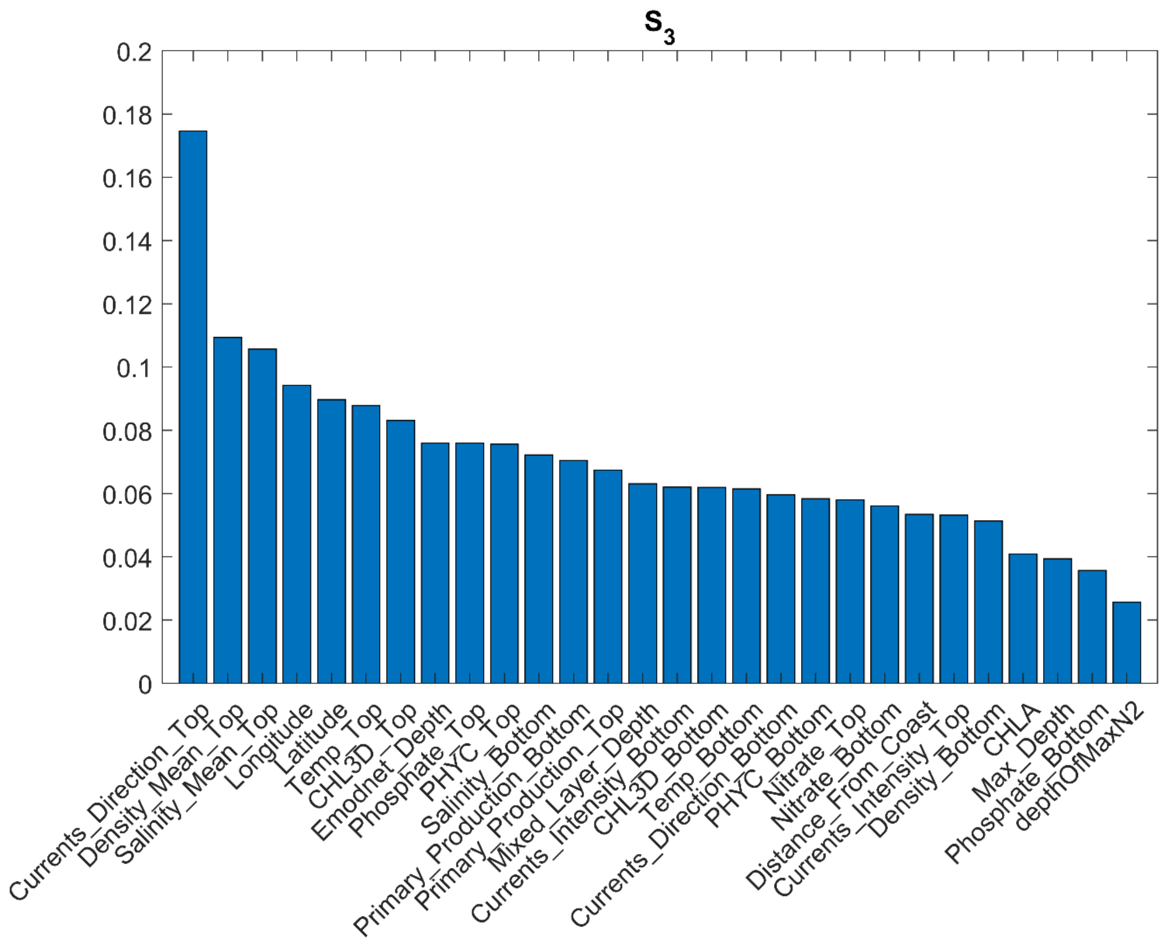

Figure S4. Features importance given by the RF model on dataset S_3.

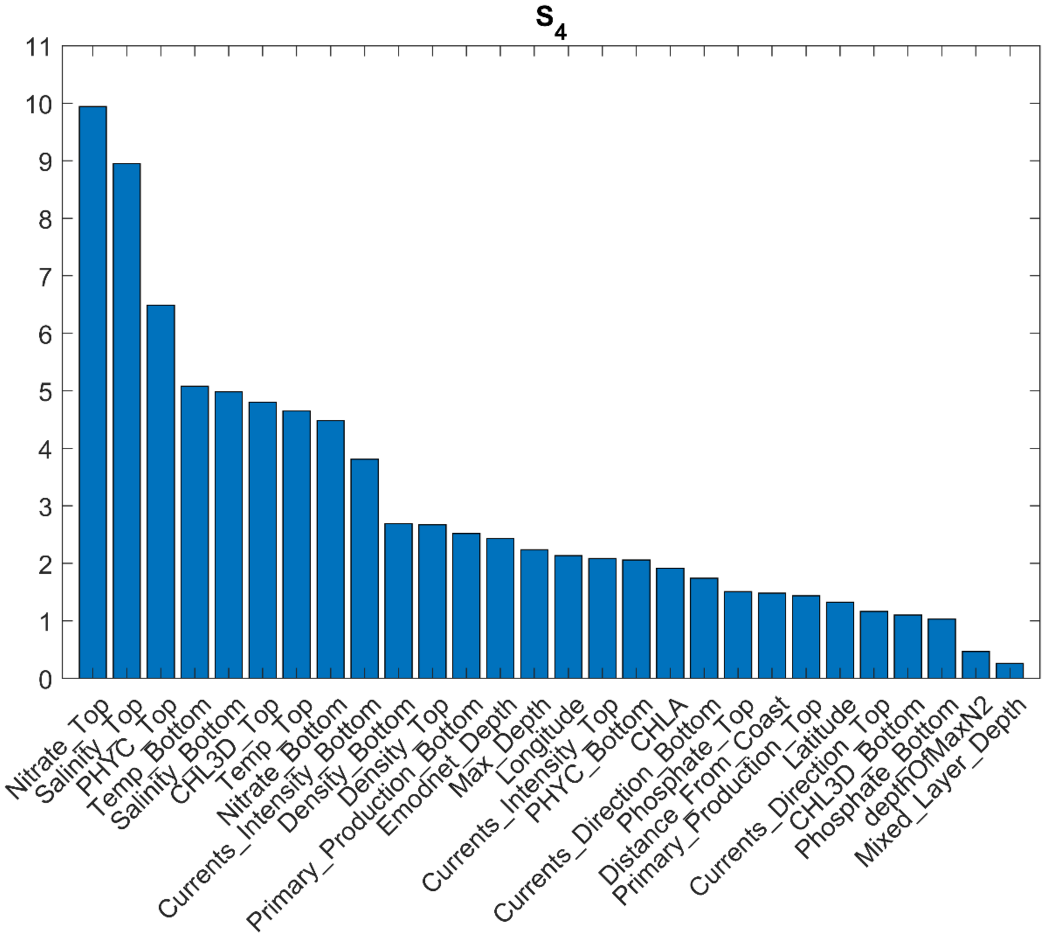

Figure S5. Features importance given by the RF model on dataset S_4.

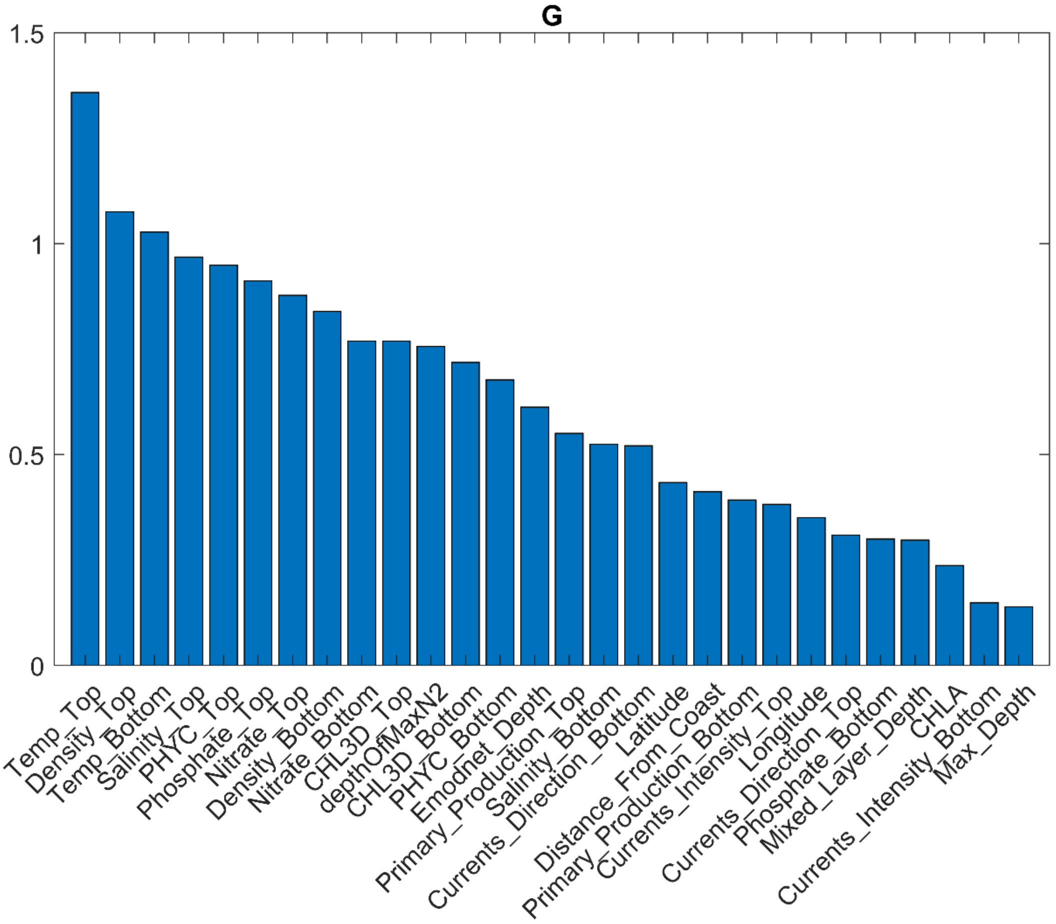

Figure S6. Features importance given by the RF model on dataset G.

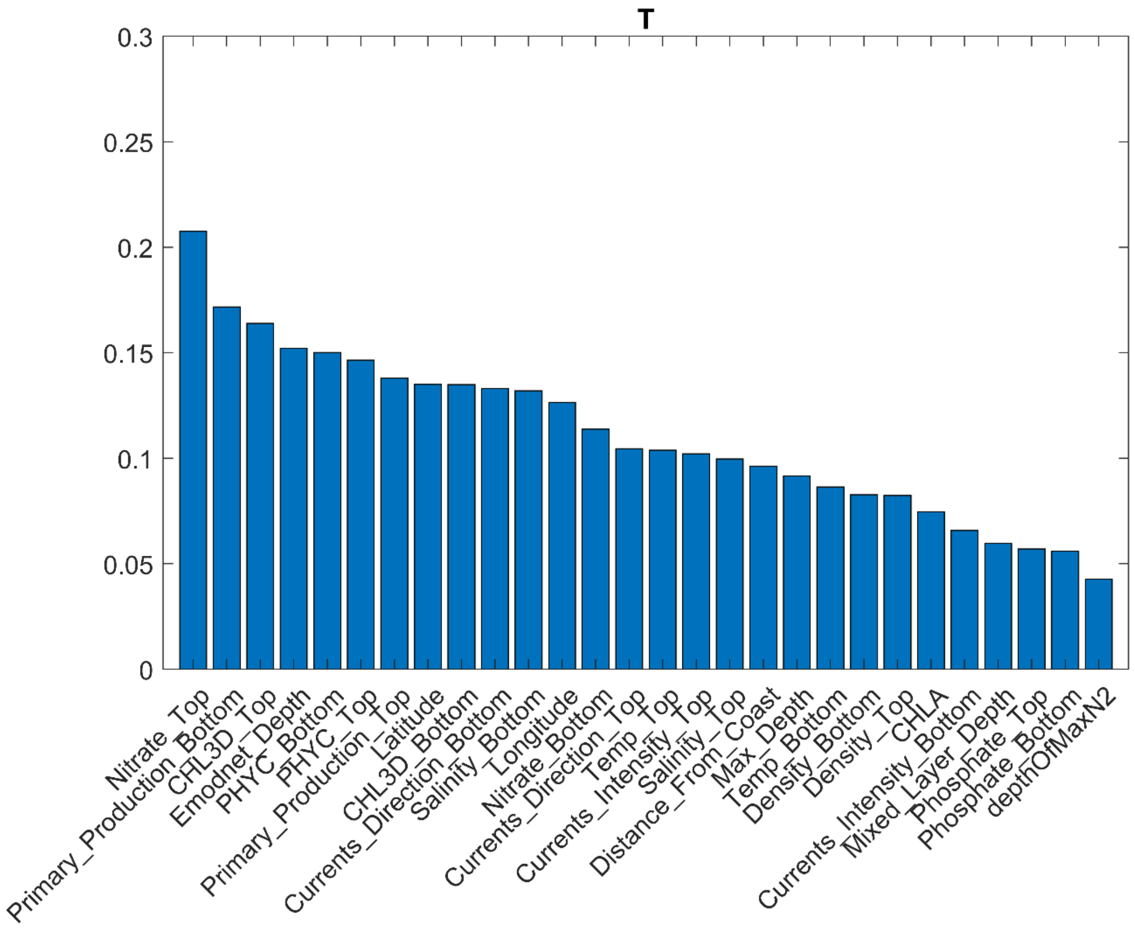

Figure S7. Features importance given by the RF model on dataset T.

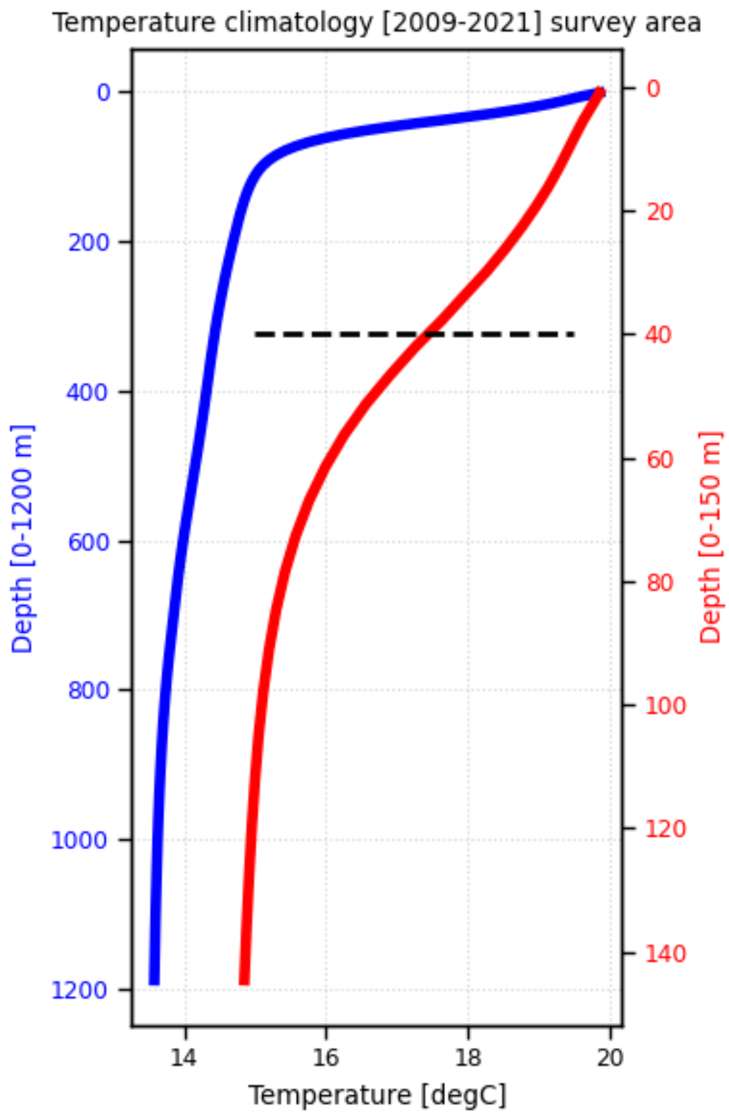

Figure S8. Average temperature profile in the study area for the period 2009-2021.

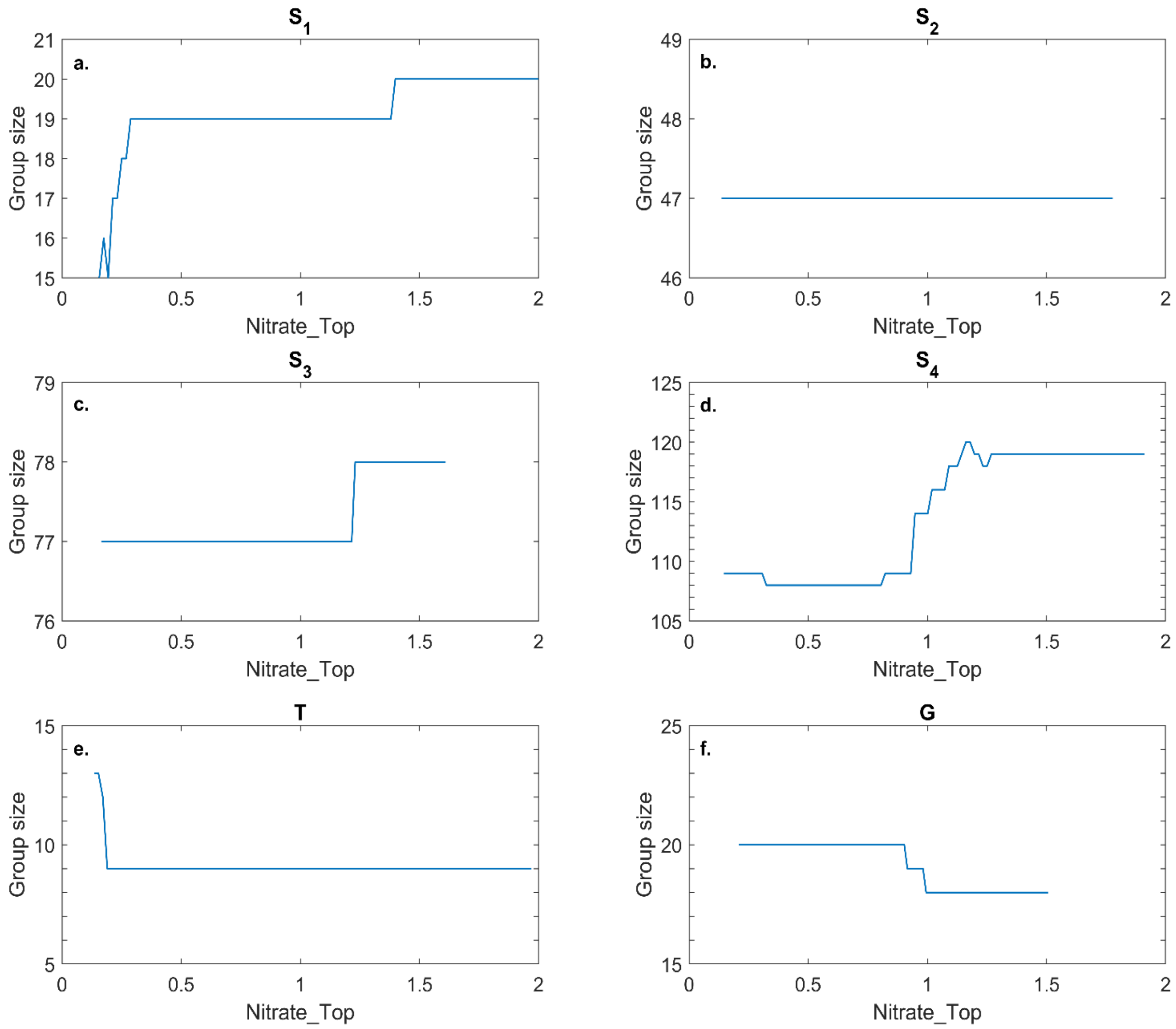

Figure S9. Partial dependence plots of group size *versus top* nitrate concentrations on datasets S_1 (a), S_2 (b), S_3 (c), S_4 (d), G (e) and T (f). The Y-axis of each plot indicates the predicted group size. The X-axis indicates the Nitrate_Top feature.

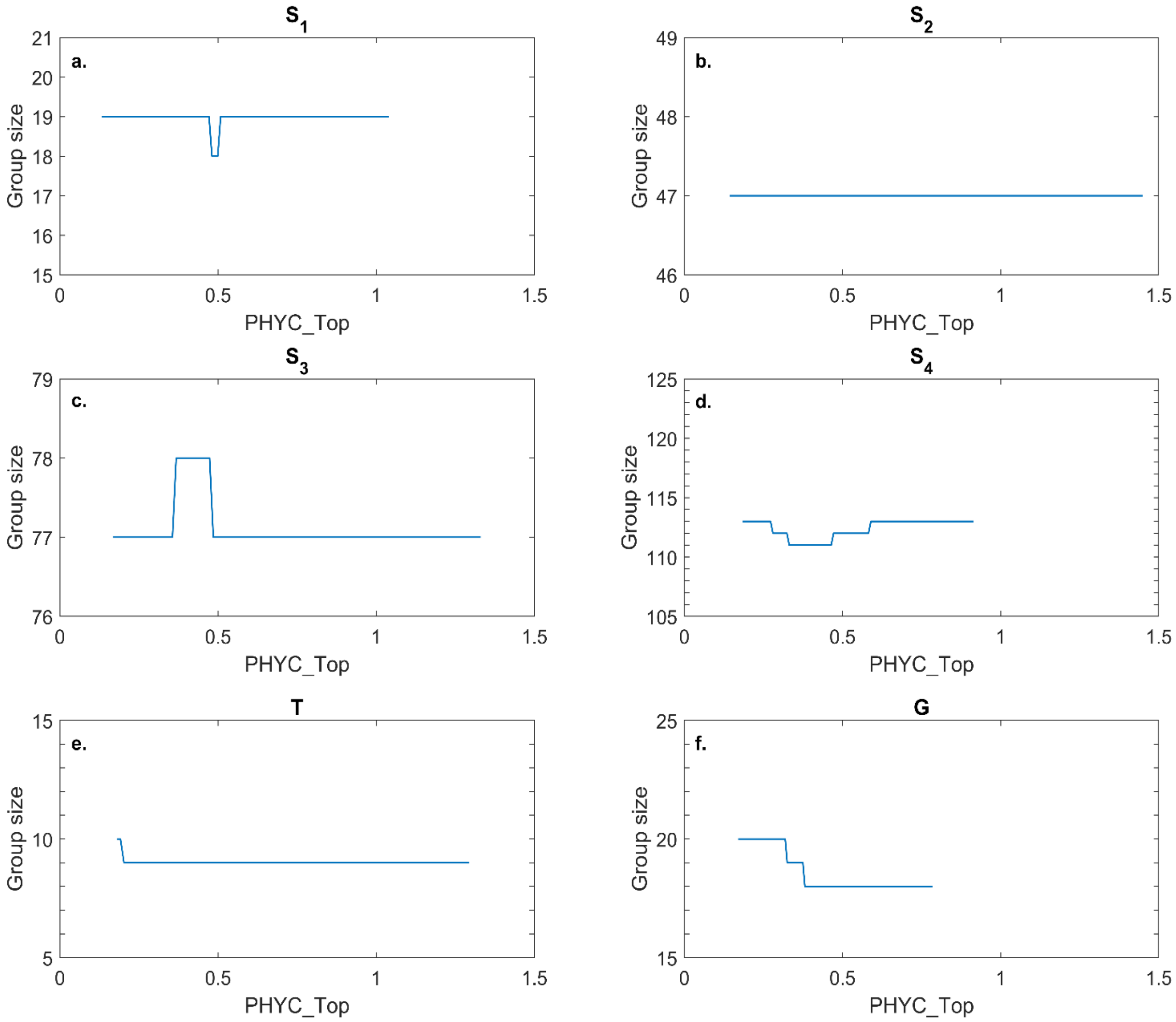

Figure S10. Partial dependence plots of group size *versus* top phytoplankton carbon biomass concentrations on datasets S_1 (a), S_2 (b), S_3 (c), S_4 (d), G (e) and T (f). The Y-axis of each plot indicates the predicted group size. The X-axis indicates the PHYC_Top feature.

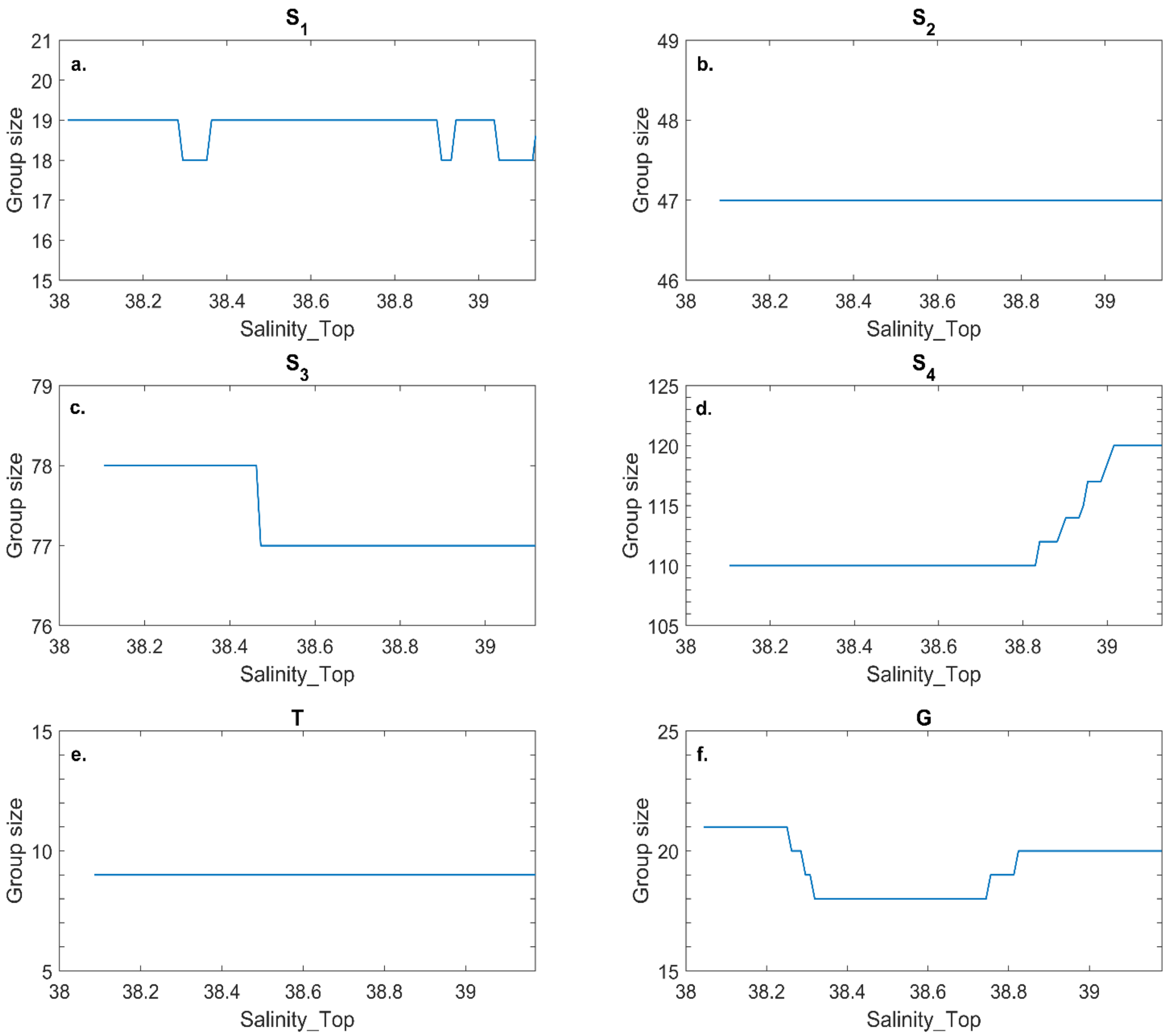

Figure S11. Partial dependence plots of group size *versus* top salinity on datasets S_1 (a), S_2 (b), S_3 (c), S_4 (d), G (e) and T (f). The Y-axis of each plot indicates the predicted group size. The X-axis indicates the Salinity_Top feature.

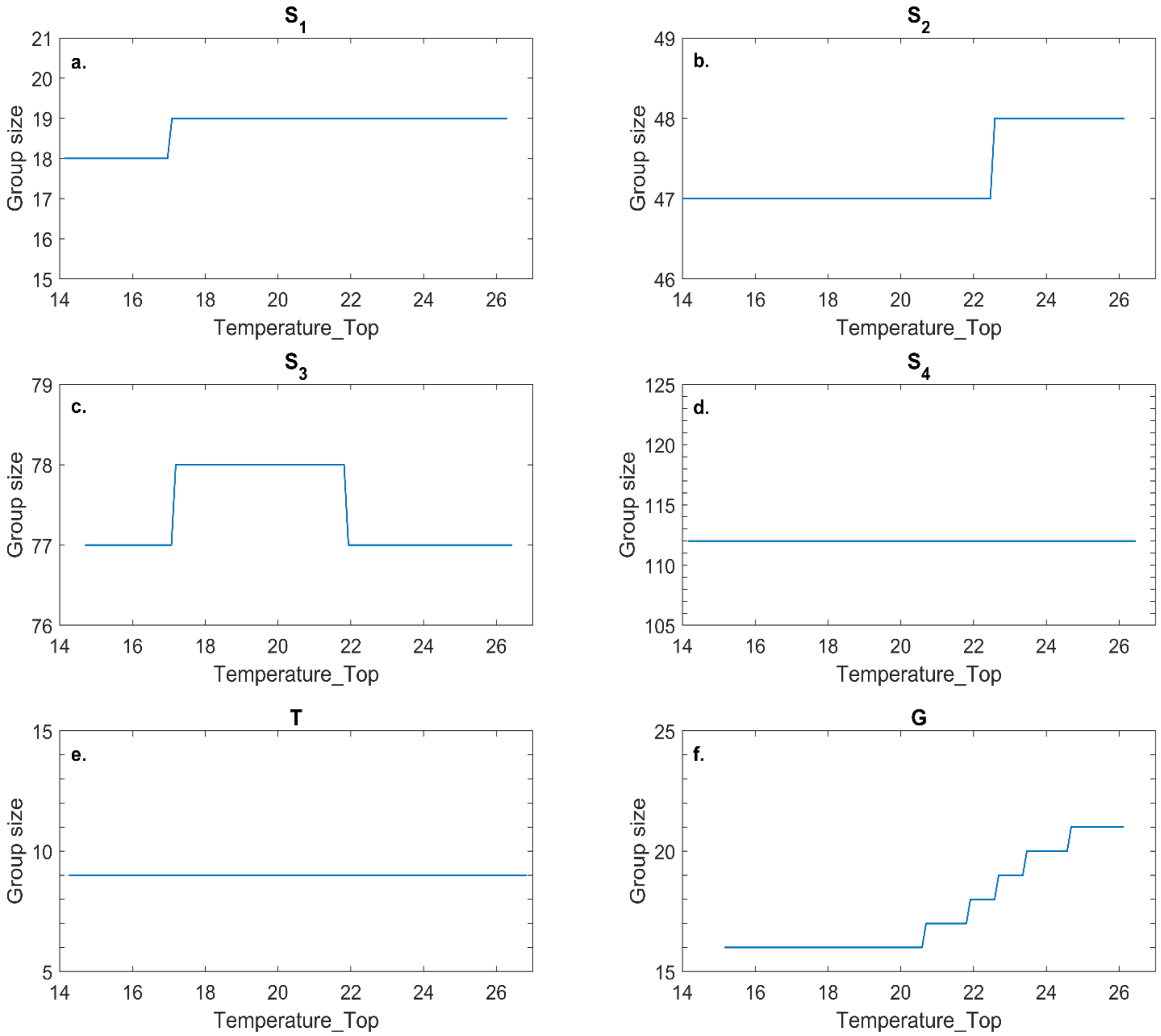

Figure S12. Partial dependence plots of group size *versus* top temperature on datasets S_1 (a), S_2 (b), S_3 (c), S_4 (d), G (e) and T (f). The Y-axis of each plot indicates the predicted group size. The X-axis indicates the Temperature_Top feature.
